# Localized conditional induction of brain arteriovenous malformations in a mouse model of hereditary hemorrhagic telangiectasia

**DOI:** 10.1007/s10456-023-09881-w

**Published:** 2023-05-23

**Authors:** Lea Scherschinski, Chul Han, Yong Hwan Kim, Ethan A. Winkler, Joshua S. Catapano, Tyler D. Schriber, Peter Vajkoczy, Michael T. Lawton, S. Paul Oh

**Affiliations:** 1 Department of Translational Neuroscience, Barrow Aneurysm and AVM Research Center, St. Joseph’s Hospital and Medical Center, Barrow Neurological Institute, 350 W. Thomas Rd., Phoenix, AZ 85013 USA; 2 Department of Neurosurgery, St. Joseph’s Hospital and Medical Center, Barrow Neurological Institute, Phoenix, AZ USA; 3 Department of Neurosurgery, Charité – Universitätsmedizin Berlin corporate member of Freie Universität Berlin, Humboldt-Universität zu Berlin, Berlin Institute of Health, Berlin, Germany

**Keywords:** Activin receptor-like kinase 1, Brain arteriovenous malformation, Hemorrhage, Hereditary hemorrhagic telangiectasia, Magnetic resonance imaging, Mouse model, Stereotaxic

## Abstract

**Background:**

Longitudinal mouse models of brain arteriovenous malformations (AVMs) are crucial for developing novel therapeutics and pathobiological mechanism discovery underlying brain AVM progression and rupture. The sustainability of existing mouse models is limited by ubiquitous Cre activation, which is associated with lethal hemorrhages resulting from AVM formation in visceral organs. To overcome this condition, we developed a novel experimental mouse model of hereditary hemorrhagic telangiectasia (HHT) with CreER-mediated specific, localized induction of brain AVMs.

**Methods:**

Hydroxytamoxifen (4-OHT) was stereotactically delivered into the striatum, parietal cortex, or cerebellum of R26^CreER^; *Alk1*^2f/2f^ (*Alk1*-iKO) littermates. Mice were evaluated for vascular malformations with latex dye perfusion and 3D time-of-flight magnetic resonance angiography (MRA). Immunofluorescence and Prussian blue staining were performed for vascular lesion characterization.

**Results:**

Our model produced two types of brain vascular malformations, including nidal AVMs (88%, 38/43) and arteriovenous fistulas (12%, 5/43), with an overall frequency of 73% (43/59). By performing stereotaxic injection of 4-OHT targeting different brain regions, *Alk1*-iKO mice developed vascular malformations in the striatum (73%, 22/30), in the parietal cortex (76%, 13/17), and in the cerebellum (67%, 8/12). Identical application of the stereotaxic injection protocol in reporter mice confirmed localized Cre activity near the injection site. The 4-week mortality was 3% (2/61). Seven mice were studied longitudinally for a mean (SD; range) duration of 7.2 (3; 2.3−9.5) months and demonstrated nidal stability on sequential MRA. The brain AVMs displayed microhemorrhages and diffuse immune cell invasion.

**Conclusions:**

We present the first HHT mouse model of brain AVMs that produces localized AVMs in the brain. The mouse lesions closely resemble the human lesions for complex nidal angioarchitecture, arteriovenous shunts, microhemorrhages, and inflammation. The model’s longitudinal robustness is a powerful discovery resource to advance our pathomechanistic understanding of brain AVMs and identify novel therapeutic targets.

**Supplementary information:**

The online version contains supplementary material available at 10.1007/s10456-023-09881-w.

## Introduction

Brain arteriovenous malformations (AVMs) represent a leading cause of hemorrhagic stroke in young adults [1, 2]. The AVM angioarchitecture is complex and comprises a high-pressure, low-resistance nidus of dysplastic blood vessels prone to spontaneous rupture and intracranial hemorrhage [3, 4]. Hereditary hemorrhagic telangiectasia (HHT, Osler–Weber–Rendu syndrome) is an autosomal-dominant vascular disorder caused by heterozygous loss-of-function mutations in activin receptor-like kinase 1 (*ALK1*), endoglin (*ENG*), or *SMAD4* [5, 6]. Pathognomic characteristics of HHT include the development of mucocutaneous telangiectasias and AVMs in the lungs, liver, and brain, with up to 20% of HHT patients exhibiting cerebral vascular malformations [6, 7].

Mouse models have provided valuable insights into the underlying genetic mechanisms of HHT and have served as the foundation for genetic-based approaches to generate animal models that simulate human conditions [8]. In the past, animal models of HHT have been engineered for homozygous or heterozygous *Alk1* or *Eng* gene knockout. This approach is limited by high rates of in utero mortality or low rates of AVM formation that disrupt AVM reproduction and preclude longitudinal studies [9, 10]. Recent advances in HHT studies have targeted Cre-mediated time-dependent or tissue-specific deletions (or both) of *Eng*, *Alk1*, or *Smad4*, either through systemic Cre activation or focal delivery of Cre-expressing adenoviral vectors [11–15]. Although these models have been instrumental in advancing our mechanistic understanding of disease pathology, several factors limit the value of current mouse models of HHT. These factors include a lack of specificity, with development of randomly located AVMs in the body [12], high rates of early lethality due to gastrointestinal hemorrhage [12], or the lack of a true vascular nidus [14, 16].

To address these limitations, we developed a novel experimental mouse model of HHT using targeted local drug delivery to induce brain AVMs in specific brain regions of *Alk1*-inducible knockout (*Alk1*-iKO) mice. Our model produced brain AVMs with high consistency and close resemblance to the phenotypical hallmarks of human pathology while obviating the off-target effects commonly associated with systemic HHT gene deletion. Furthermore, the model’s ability to recapitulate disease progression over time makes it an invaluable tool for understanding the pathogenesis of brain AVMs and accelerating the development of new and effective therapeutics for individuals affected by HHT.

## Methods

Detailed experimental procedures are available in the supplemental methods.

## Results

### Localized induction of brain AVMs

**Fig. 1:**
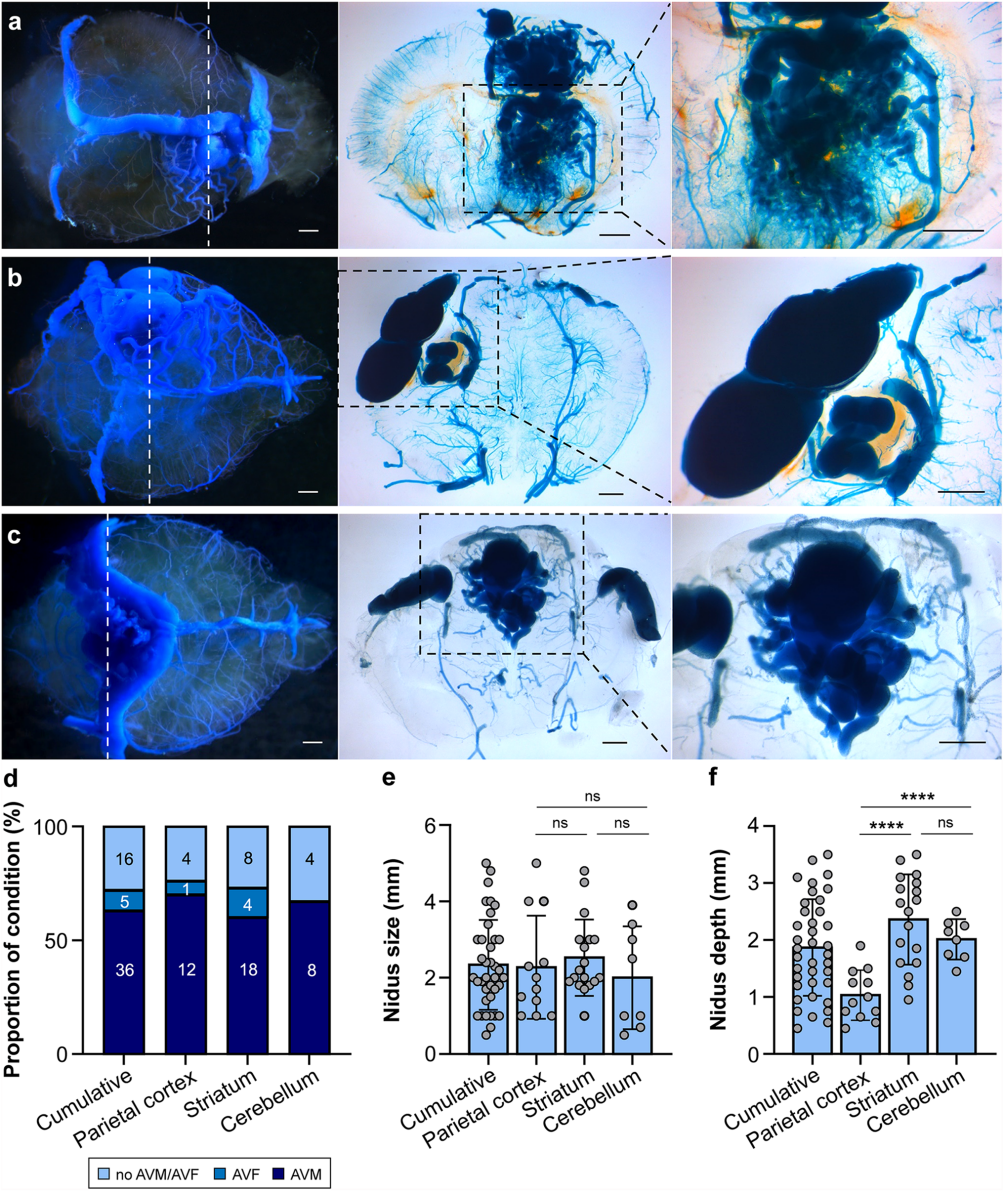
Localized induction of brain AVMs in a mouse model of HHT. Representative microscopy imaging showing latex dye-perfused brains with brain AVMs in the right-side striatum (*white dashed lines* show slice location) (**a**), left-side parietal cortex (**b**), and midline cerebellum (**c**). Whole brain (*left*) and low-magnification (*center*; *black dashed lines* indicate areas of magnification) and high-magnification (*right*) coronal section images. Bar, 1 mm. **d** Bar graph representing frequencies of brain AVM development after stereotaxic intracerebral injection of 4-OHT into the striatum, parietal cortex, and cerebellum. Mean (SD). Bar graphs representing the nidus size (**e**) and lesion depth (**f**) after stereotaxic intracerebral injection of 4-OHT into the striatum, parietal cortex, and cerebellum. Mean (SD), ANOVA. ****p < 0.001; *ns* not significant

To develop a novel mouse model of brain AVMs with targeted intracerebral lesion induction, we performed stereotaxic intracerebral injection of 4-hydroxytamoxifen (4-OHT) on postnatal day 1 (P1) in *Alk1*-iKO mice and their control littermates. We evaluated them for vascular lesions 3–4 weeks postinjection using latex dye perfusion. We targeted multiple mouse brain regions with distinct spatial distributions to investigate the spatial relationship of vascular lesion formation relative to the injection site. We targeted the right-hemispheric striatum, the left-hemispheric parietal cortex, and the midline cerebellum (Supplemental Table 1). Stereotaxic injection of 4-OHT into the brain target regions in *Alk1*-iKO mice produced brain vascular lesions in the striatum in 73% (nidal AVM, 82% [18/22]; arteriovenous fistula [AVF], 18% [4/22]) of brains, in the parietal cortex in 76% (nidal AVM, 92% [12/13]; AVF 8% [1/13]), and in the cerebellum in 67% (nidal AVM, 100% [8/8]) (Fig. 1a–d). Among all *Alk1*-iKO mice injected intracerebrally with 4-OHT (*n* = 59, female sex, 33 [56%]), 73% (43/59) developed brain vascular malformations, including nidal brain AVMs (88%, 38/43) and AVFs (12%, 5/43). 4-OHT—injected CreER-negative littermates served as negative controls and did not form brain vascular lesions (*n* = 40) (Supplemental Fig. 1). The 4-week mortality was 3% (2/61). Both mice developed extensive intracranial hemorrhages from spontaneous brain AVM rupture.

To characterize the vascular lesions produced by our technique, we measured the nidus size and lesion depth in coronal brain tissue sections (Fig. 1e and f). The cumulative mean (SD; range) nidus size of all brain AVMs was 2.33 (1.16; 0.5–4.8) mm, and the mean (SD; range) lesion depth was 1.85 (0.86; 0.45–3.5) mm. Brain AVMs were largest in the striatum with a mean (SD) nidus size of 2.53 (1.0) mm, followed by parietocortical AVMs at 2.28 (1.35) mm and cerebellar AVMs with 1.85 (1.27) mm (Fig. 1e). The mean (SD) lesion depth varied significantly between striatal and parietocortical brain AVMs (2.36 [0.79] mm vs. 1.03 [0.44] mm, *p* < 0.001) and between cerebellar and parietocortical AVMs (1.93 [0.35] mm vs. 1.03 [0.44] mm, *p* < 0.001) (Fig. 1f). Brain AVMs formed in the target injection region with 98% (40/41) accuracy; thus, all brain AVMs developed at or near the injection site and in the intended hemisphere (parietal cortex, left hemisphere; striatum, right hemisphere) or along the midline (cerebellum). One mouse was injected into the midline cerebellum but was identified as having formed a brain AVM in the right anterior hemisphere in subcortical structures.

Most brain AVMs were associated with various degrees of intracranial microhemorrhage. Five mice presented with hydrocephalus clinically evident as reduced mobility, apathy, and increased head circumference. The presence of hydrocephalus was confirmed in all 5 after perfusion with latex dye (Supplemental Fig. 2a). One mouse in the longitudinal analysis gradually developed radiographic signs of hydrocephalus starting at 5 months of age without clinical evidence of increased intracranial pressure (Supplemental Fig. 2b).

### Validation of injection site-specific genetic recombination

Using our stereotaxic injection protocol in R26^CreER/mTmG^;*Alk1*^2f/2f^ reporter mice, we sought to visualize the extent of genetic recombination induced in the mouse brain by the technique. In mT/mG reporter mice, mT labeling (TdTomato) is constitutively expressed and replaced with mG labeling (GFP fluorescence) upon activation of Cre. Among 6 mutant R26^CreER/mTmG^;*Alk1*^2f/2f^ mice that received a single dose of 4-OHT into the left parietal cortex, 4 developed brain AVMs, and all had Cre-mediated mG labeling adjacent to the lesion margin in the brain cortex (Supplemental Fig. 3). GFP fluorescence was not detectable in 2 mutants that had not formed brain AVMs, suggesting that no genetic recombination occurred in mice that did not form brain AVMs.

### Timing of brain AVM development

**Fig. 2:**
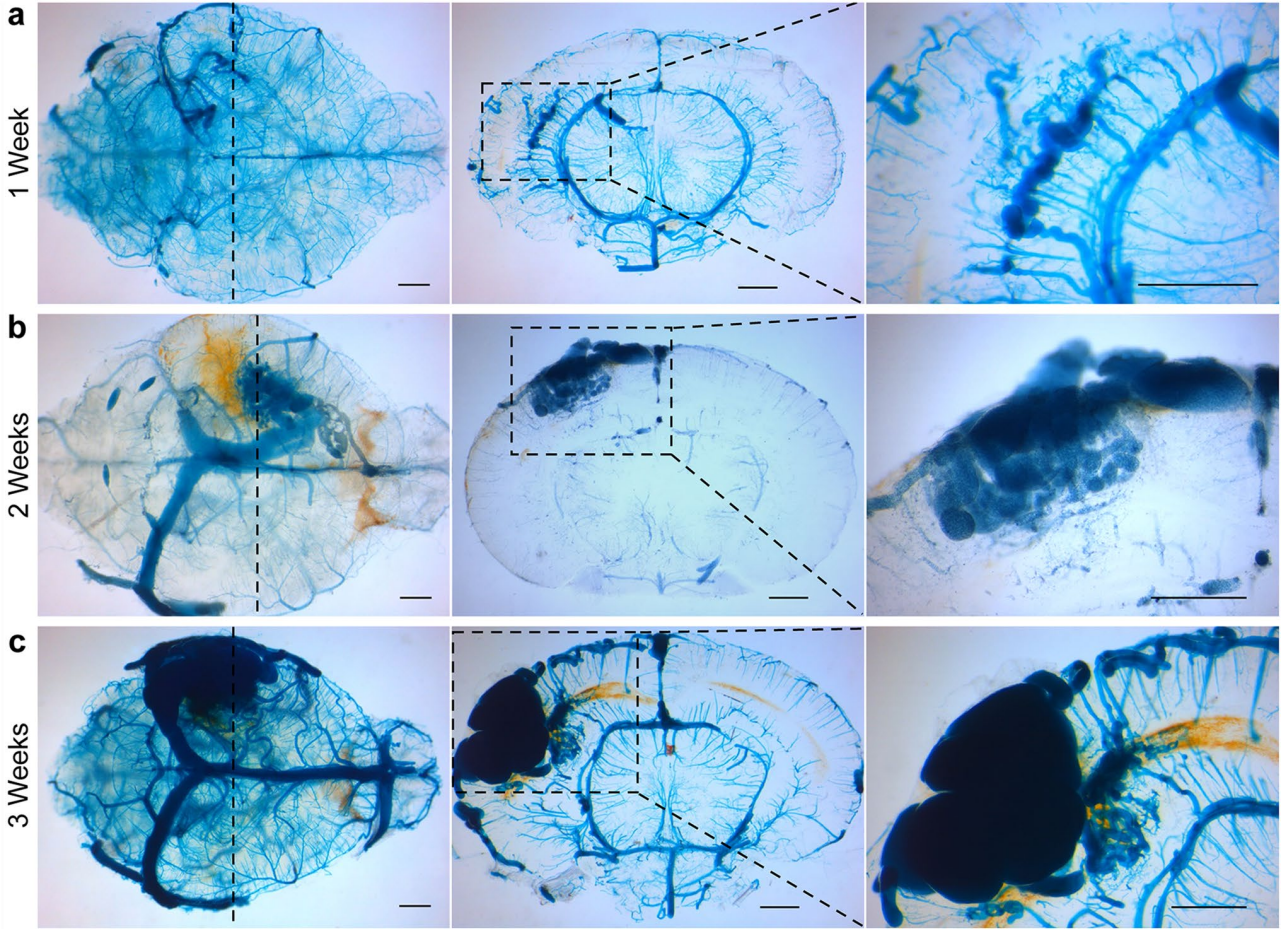
Timing of brain AVM development after stereotaxic intracerebral injection of 4-OHT in *Alk1*-inducible knockout mice. **a** Microscopy images of latex dye-perfused brains showing a brain AVM in the parietal cortex 7 days postinjection (*n* = 3). **b** Microscopy images showing a brain AVM in the parietal cortex 14 days postinjection (*n* = 2). **c** Microscopy images showing a brain AVM in the parietal cortex 21 days postinjection (*n* = 5). Whole brain (*left; dashed line* shows location) and low-magnification (*center*; *black outlines* indicate area of magnification) and high-magnification (*right*) coronal section images. Bar, 1 mm

Although several mouse models simulate adult-onset brain AVM formation [14, 15], modeling brain AVMs in the postnatal period is less established. We sought to investigate the timing of brain AVM development in this model. Brain AVMs started forming as early as 1 week postinjection of 4-OHT and displayed a premature phenotype with tortuous vessel formation while remaining small (Fig. 2a). In contrast, brain AVMs analyzed 2 and 3 weeks postinjection gradually increased in size and displayed a mature and complex nidal phenotype (Fig. 2b and c).

### Intracerebral versus systemic conditional gene deletion

The systemic activation of Cre in conditional knockout mice results in micro- and macrohemorrhaging from AVMs in internal organs [12, 17]. To showcase the effect differences of intracerebral versus systemic Cre activation, we treated neonatal *Alk1*-iKO mice (P1) with tamoxifen by intragastric injection (50 mg/kg body weight). The intragastric application of tamoxifen in neonatal *Alk1*-iKO mice was associated with an immediate 100% mortality rate within the first week of life in all mice (*n* = 5), exemplifying the potency of ubiquitous *Alk1* deletion through systemic Cre activation. Therefore, we adapted our experimental design to administer tamoxifen to 4-week-old *Alk1*-iKO mice by intraperitoneal injection (200 mg/kg body weight). 1 week posttreatment, the hemoglobin levels of systemically tamoxifen-treated *Alk1*-iKO mice were analyzed and compared to stereotactically 4-OHT-injected *Alk1*-iKO mice. Systemically treated *Alk1*-iKO mutants had significantly decreased hemoglobin levels compared to control mice (15.98 g/dL vs. 5.67 g/dL, *p* < 0.001) and compared to stereotactically treated *Alk1*-iKO mutants (13.31 g/dL vs. 5.67 g/dL, *p* < 0.001) (Supplemental Fig. 4a). Mean (SD) hemoglobin levels were not statistically different between stereotactically injected *Alk1*-iKO mutant and control mice (13.31 g/dL vs. 13.97 g/dL, *p* = 2.0). Based on the hemoglobin analysis, stereotaxic injection of 4-OHT was restricted to producing AVMs in the brain, whereas the systemic injection of tamoxifen was associated with the development of AVMs in the ears, cecum, and stomach (Supplemental Fig. 4b).

### Longitudinal HHT mouse model of brain AVMs

**Fig. 3:**
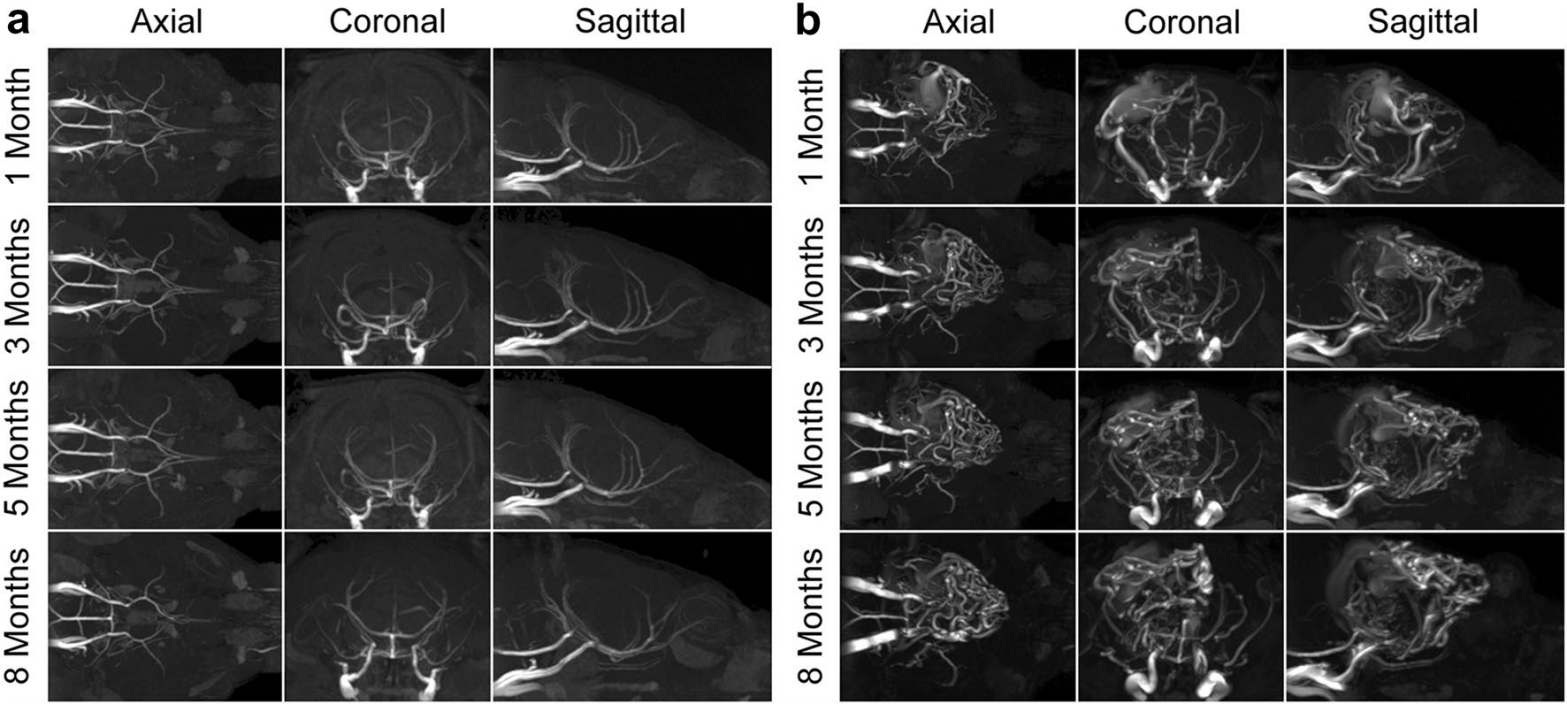
Longitudinal MRA of a brain AVM in an HHT mouse model. **a** MRA imaging showing axial (*left*), coronal (*center*), and sagittal (*right*) views of a control mouse brain with normal angioarchitecture at 1, 3, 5, and 8 months. **b** MRA imaging showing axial (*left*), coronal (*center*), and sagittal (*right*) views of a large nidal brain AVM in the left-side parietal cortex at 1, 3, 5, and 8 months

After stereotaxic injection of 4-OHT into the left parietal cortex, 7 *Alk1*-iKO mice were identified as having developed vascular lesions on 1-month magnetic resonance angiography (MRA) imaging and were included for longitudinal analysis (Table 1). Control mice displayed normal cerebrovasculature (Fig. 3a). All brain AVMs developed in the left parietal cortex. Four mice formed large nidal AVMs, 1 formed a small nidal AVM, 1 an AVF, and 1 an extracranial–intracranial AVM. One mouse with a large nidal AVM died at 2 months of age, likely due to spontaneous AVM rupture; 1 mouse developed aggressive behavior warranting early termination; and a 9-month-old mouse with a large nidal AVM developed progressive face swelling, likely due to venous congestion. Four of 7 mice were alive at the end of the mean (SD; range) study period of 7.2 (3; 2.3–9.5) months. However, after accounting for the event of necessary early termination, the long-term survival rate of this model was 75% (4/5) at 9 months. Brain AVM nidi were grossly stable on serial 3D time-of-flight MRA, with radiographic evidence of remodeling between 1 and 3 months after the onset of brain AVM development (Fig. 3b).

**Table 1:** Analysis of 3D MR gradient echo flow compensation angiograms

Mouse no.	Vascular lesion	Age (months)	Sex	Target location	Nidus size (mm)	Compactness
1	AVM	9.4	M	L parietal cortex	4.54	Compact
2	AVM	5.1	M	L parietal cortex	4.80	Compact
3	AVM	9.2	F	L parietal cortex	5.21	Compact
4	AVM	9.4	M	L parietal cortex	2.56	Compact
5	AVM	2.3	F	L parietal cortex	4.47	Diffuse
6	AVM	5.1	M	L parietal cortex	3.90	Diffuse
7	AVF	9.4	M	L parietal cortex	N/A	N/A

### Histological characterization of brain AVMs

**Fig. 4:**
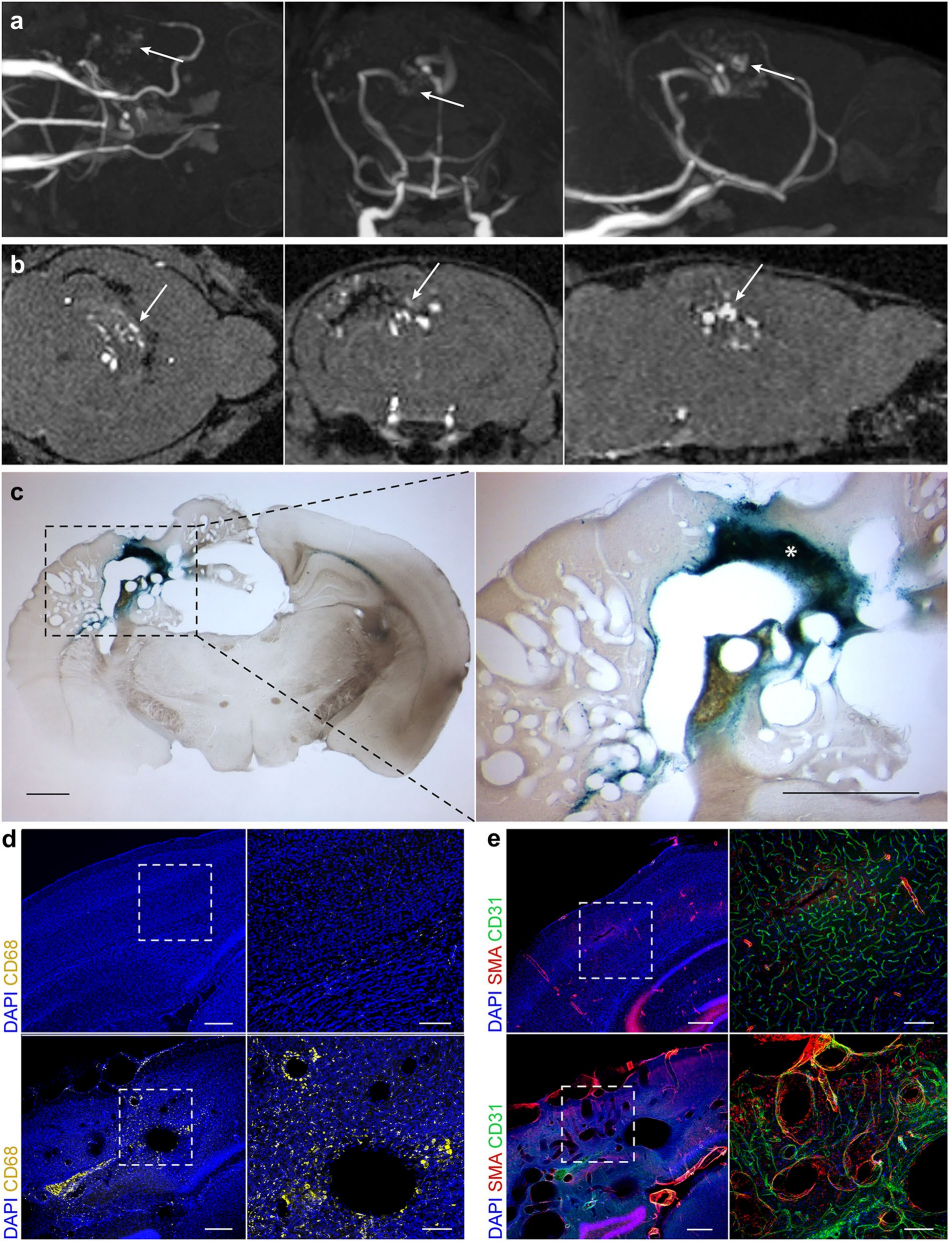
Histological analysis of brain AVMs in *Alk1*-inducible knockout mice induced by stereotaxic intracerebral injection of 4-OHT. **a** 3-Dimensional and 2-dimensional **b** time-of-flight MRA imaging showing axial (*left*), coronal (*center*), and sagittal (*right*) views of a brain AVM at 3 months of age. *White arrows* indicate location of the brain AVM. **c** Microscopy images of coronal mouse brain tissue section after Prussian blue staining showing microhemorrhages surrounding the nidal vasculature. *Area of ferric iron deposition. Low-magnification (*left*; *dashed black outlines* indicate area of magnification; bar 1 mm) and high-magnification (*right*, bar 1 mm) coronal section images. **d** Coronal mouse brain tissue section of control (*top* panels) and *Alk1*-inducible knockout (*bottom* panels) mouse after immunostaining for monocytes/macrophages (CD68). Low-magnification (*left*; *white outlines* indicate area of magnification, bar 250 μm) and high-magnification (*right*, bar 100 μm) coronal section images. **e** Coronal mouse brain tissue section of control (*top* panels) and *Alk1*-inducible knockout (*bottom* panels) mouse after immunostaining for endothelial cells (CD31) and alpha-SMA. Low-magnification (*left*; *white outlines* indicate area of magnification, bar 250 μm) and high-magnification (*right*, bar 100 μm) coronal section images

The morphological hallmarks of human brain AVMs include the complexity of nidus formation, recurrent microhemorrhages, and invasion of immune cells leading to focal inflammation [18]. To confirm the presence of these hallmarks in our mouse model, we performed immunohistochemical staining on a 3-month-old mouse brain with an AVM confirmed with 3D time-of-flight MRA (Fig. 4a) and 2D MRA imaging (Fig. 4b). Prussian blue staining showed microhemorrhages centered in the brain AVM nidus and surrounding this vasculature (Fig. 4c). Microhemorrhages correlated with a diffuse pattern of CD68-positive cells surrounding the nidal vessels, which were not detected in control brains (Fig. 4d). Multifluorescent immunostaining for endothelial cells (CD31) and smooth muscle cells (SMA) showed complex nidal vasculatures in coronal brain tissue sections, which were absent in controls (Fig. 4e).

## Discussion

Brain AVMs are an important cause of hemorrhagic stroke, with limited availability of safe and effective, noninvasive treatment options to prevent them from bleeding [19, 20]. Our understanding of the detailed mechanisms underlying disease progression remains enigmatic. There is a lack of high-fidelity mouse models that (1) produce brain AVMs with high efficiency and without lethal off-target effects, (2) produce brain AVMs with a high degree of similarity with the human disease pathology, and (3) permit longitudinal disease modeling. We present the first HHT mouse model that allows for targeted induction of brain AVMs in transgenic mice using stereotaxic intracerebral injection of 4-OHT. The results demonstrate a robust longitudinal mouse model that closely mimics the pathobiological hallmarks of human brain AVMs, including microhemorrhage, immune cell invasion, nidus formation, and spontaneous rupture.

### Mouse model of familial brain AVMs in context

Our group previously developed a longitudinal HHT mouse model using *Tagln*-Cre;*Alk1*^2f/2f^ transgenic mice and characterized the vascular lesions histologically and radiographically [12]. This model is based on the conditional genetic deletion of *Alk1* during embryogenesis, which led to the development of nidal brain AVMs in 40% of mice, AVFs in 18%, and no vascular lesions in 42%. On high-resolution digital subtraction angiography, the model recapitulated the radiographic key features of human disease, including early arteriovenous shunting, complex nidus angioarchitecture, microhemorrhages, and nidal stability for a mean duration of 9.5 months (range, 3–17 months). However, we noted a 4-week mortality of 20% and an additional 35% mortality by the age of 6 months, likely related to hemorrhages in the gastrointestinal tract, lungs, and brain. One of the key advantages of our mouse model is the reduction of mortality rates compared to traditional models. This reduction was achieved by avoiding side effects related to the systemic administration of tamoxifen, such as anemia and multisite hemorrhages due to ubiquitous Cre activation, producing improved long-term survival.

Walker et al. developed a mouse model that employed stereotaxic intracranial delivery of adenoviral vector—expressing Cre recombinase combined with an adeno-associated viral vector expressing vascular endothelial growth factor (VEGF) to generate brain AVMs in adult *Alk1*^2f/2f^ mice [14]. Unlike our model, which primarily produced a complex vascular nidus, the Walker et al. model is characterized by increased vessel density and vascular dysplasia as the primary pathological features. The phenotypical hallmark of complex nidus formation is disease-specific and essential for deciphering the cellular and molecular changes underlying altered hemodynamics in brain AVMs. These features provide accurate and reliable representations of the pathology seen in humans. Zhu et al. used CRISPR/Cas9 technology to locally introduce somatic endothelial cell-specific *Alk1* gene mutations in brains of adult wild-type mice [21]. An adeno-associated viral vector expressing VEGF was coadministered for angiogenesis in these mouse brains. The model produced arteriovenous shunts with an increased vessel dysplasia index but without a true vascular nidus.

Another approach to creating a mouse model of brain AVMs is to induce somatic activating *KRAS* mutations, which have been implicated in the pathogenesis of human sporadic brain AVMs [22–25]. The viral-mediated endothelial cell-specific overexpression of mutant KRAS^G12V^ protein generated multiple small nidi in the mouse brain [25]. These nidi were accompanied by additional characteristics, including upregulation of endogenous VEGF signaling, spontaneous multifocal intracerebral hemorrhages, neuroinflammation, and sensory, cognitive, and motor behavior dysfunction [25]. Although this approach provides valuable insights into the underlying mechanisms of sporadic brain AVMs, it might not be applicable to study the genetic interactions that occur in familial brain AVMs.

Our mouse model provides a short latency and AVM production without angiogenic stimulation or highly complex genetic engineering techniques. The vascular lesions are detectable 1 week postprocedure, with progressive maturation into complex nidal brain AVMs by 3 to 4 weeks of age, and are associated with markedly elevated neuroinflammation. These features distinguish our mouse model from others [14, 15, 21, 25–27], requiring costly genetic engineering techniques, long latency times, and additional angiogenic stimulation to induce brain AVMs.

## Technical aspects

Intracranial stereotaxic injection is a technique used to deliver substances to specific brain regions with high spatial precision. The technique has been employed in many experimental settings, including glioblastoma [28], posttraumatic epilepsy [29], multiple sclerosis [30], Alzheimer’s disease [31], and intracranial aneurysm formation and rupture [32]. The advantages of disease induction by stereotaxic injection are high precision, easy applicability, and versatility. Varying degrees of interperformer or intersubject variability may be observed [33]. The technique is relatively noninvasive, which minimizes stress on the animal and reduces the risk of periprocedural complications. The procedure was associated with a low rate of adverse outcomes, most commonly intracerebral hemorrhage due to injury of the dural venous sinus.

## Brain AVM characteristics

The model’s validity has been established through histological and radiographic characterizations, showing that the mouse brain AVMs closely mimic the characteristics and pathology of human brain AVMs. Pathological hallmarks include the formation of a vascular nidus with complex angioarchitecture and arteriovenous shunting with vessel enlargement and tortuosity, microhemorrhages with associated neuroinflammation, and longitudinal nidal stability, with features of radiographically evident remodeling during the early stages of AVM maturation [18, 34–36].

The occurrence of hydrocephalus in mice with large, hemorrhagic brain AVMs is notable. The association between hydrocephalus and brain AVMs is well established and is a common presenting symptom of human pediatric brain AVMs [37]. However, it is seen in less than 1% of adults with AVMs [38, 39]. Hydrocephalus is most often caused by intraventricular hemorrhage, which results in malabsorption-type hydrocephalus [40]. In unruptured brain AVMs, the most common pathogenic mechanisms of hydrocephalus involve mechanical obstruction by the draining vein or AVM nidus or venous congestion [37]. Because all hydrocephalic mice in this study had large nidal brain AVMs with microhemorrhages, malabsorption caused by blockage of the arachnoid granulations could be implicated in the pathogenesis of hydrocephalus, along with mechanical venous or ventricular obstruction.

## Limitations

The model’s limitations relate to technical and precision challenges. Although we demonstrated spatial lesion variability in various brain areas, we could not induce uniformly sized lesions in all brain regions. This phenomenon was likely due to the limited solubility of 4-OHT in water, making it difficult to achieve consistent and uniform dosing across all animals. Furthermore, the long-term survival might not accurately reflect the true survival rate of the model due to the small sample size and possible early terminations. Despite these limitations, the minimal rates of early lethality associated with localized Cre induction portend excellent long-term survival rates compared to other mouse models.

## Conclusion

We present the first mouse model of HHT with localized induction of brain AVMs through stereotaxic CreER-mediated genetic recombination. The model efficiently produces brain AVMs while bypassing drivers of early morbidity and mortality. The lesions closely resemble human disease pathology, including complex nidus formation with arteriovenous shunts, microhemorrhages, immune cell infiltration, and nidal stability. Notable technical advantages of this model include its easy applicability, versatility, and no need for angiogenic stimulation. The model’s capacity to reliably reproduce brain AVM in various brain regions and its robustness in recapitulating disease progression over time make it a valuable tool to reveal the pathological mechanisms underlying brain AVM formation and develop novel therapeutic strategies with accelerated translation.

### Supplementary information

Below is the link to the electronic supplementary material.

Supplementary material 1 (PDF 10238.6 kb)

## Abbreviations

AVF: Arteriovenous fistula
AVM: Arteriovenous malformation
*Alk1*: Activin receptor-like kinase 1
*Eng*: Endoglin
HHT: Hereditary hemorrhagic telangiectasia
iKO: inducible knockout
MRA: Magnetic resonance angiography
MRI: Magnetic resonance imaging
4-OHT: 4-Hydroxytamoxifen
PB: Phosphate buffer
PBS: Phosphate-buffered saline
*Smad4*: *Caenorhabditis elegans Sma* genes fused with *Drosophila* Mad4 [Mothers against decapentaplegic homolog 4]
VEGF: Vascular endothelial growth factor
