# Supplementary material for: Localized conditional induction of brain arteriovenous malformations in a mouse model of hereditary hemorrhagic telangiectasia
Source: Angiogenesis. 2023 May 23;26(4):493–503. doi: 10.1007/s10456-023-09881-w (PMC10542309; doi:10.1007/s10456-023-09881-w)
Supplement: Supplementary file 1 — Supplementary material 1 (PDF 10238.6 kb) [file 10456_2023_9881_MOESM1_ESM.pdf]

Supplemental Materials

Supplemental Methods

Supplemental Table

Supplemental Figures 1-4

SUPPLEMENTAL METHODS

Transgenic Mice

All animal procedures were reviewed and approved by the Barrow Neurological Institute and St. Joseph's Hospital and Medical Center Institutional Animal Care and Use Committee (Protocol number: 573). Data reporting was performed in accordance with the ARRIVE 2.0 guidelines. As previously described,[1] *Alk1*-conditional knockout alleles were generated by flanking exon 5 of the *Alk1* gene using specific loxP sites. Cre-mediated excision of the floxed sequence causes the excision of exon 5, which encodes the transmembrane domain of the ALK1 receptor. $R26^{CreER/+}$ mice (The Jackson Laboratory, stock number: 008463) were crossed with $Alk1^{2f/2f}$ mice to establish the $R26^{CreER/+}$;$Alk1^{2f/2f}$ mouse strain used in this study and subsequently referred to as *Alk1*-inducible knockout (*Alk1*-iKO) mice. A dual-fluorescent Cre reporter mouse line ($R26^{CreER/mTmG}$;$Alk1^{2f/2f}$) was established by crossing $R26^{mTmG/+}$ reporter mice (The Jackson Laboratory, stock number: 007576) with $R26^{CreER/+}$;$Alk1^{2f/2f}$ mice to detect Cre-mediated recombination sites. Mice were on mixed 129Sv/C57BL6 hybrid genetic backgrounds. Both male and female mice were included and randomized.

Stereotaxic Intracerebral Injection of Hydroxytamoxifen

4-Hydroxytamoxifen (4-OHT, Sigma-Aldrich, St. Louis, MO, USA, catalog number: H7904) was dissolved in dimethyl sulfoxide (DMSO, Sigma-Aldrich) and diluted to a final concentration of 20 mM. On postnatal day 1, neonates were anesthetized using cryoanesthesia and placed into an in-house 3D-printed mouse neonatal stereotaxic adapter[2] secured in a stereotaxic frame (World Precision Instruments, Sarasota, FL, USA). A total volume of 0.5 µL 4-OHT was delivered into the brain target regions through a 30-gauge Hamilton syringe (Hamilton

Company, Reno, NV, USA, model number: 80008). Supplemental Table 1 shows the stereotaxic coordinates used to deliver 4-OHT into the striatum, parietal cortex, and cerebellum of neonatal mice on postnatal day 1 and were established based on previous reports.[3, 4] The exclusion criteria were immediate periprocedural adverse events, any procedure-related complications compromising the normal mouse behavior, or a loss of body weight >20%.

Systemic Injection of Tamoxifen

Tamoxifen (Sigma Aldrich, catalog number: T5648) was dissolved in corn oil at 25 mg/mL and was administered intragastrically to neonatal mice (50 mg/kg body weight) and intraperitoneally to adult mice (200 mg/kg body weight).

Latex Dye Perfusion

Mice were anesthetized intraperitoneally with ketamine hydrochloride (100 mg/kg body weight) and xylazine (10 mg/kg body weight). After sequential whole-body perfusion with a vessel dilator (20% heparin [50 units/mL], 1% papaverine, and 0.2% sodium nitroprusside in phosphate-buffered saline [PBS]) and 10% formalin, blue latex dye (15 μL/g body weight; VWR International, Radnor, PA, USA) was injected into the left cardiac ventricle to perfuse the cerebrovasculature. After overnight fixation in 10% formalin, brains were extracted from the cranium, dehydrated in methanol, and cleared in organic solvents (benzyl alcohol/benzyl benzoate, 1:1; Sigma-Aldrich).[5] Coronal brain sections were obtained using an adult mouse brain slicer matrix with 1.0 mm section slice intervals (Zivic Instruments, Pittsburgh, PA, USA, model number: BSMAS001-1). Images were captured using the high-resolution Leica MZ8

Stereomicroscope with a charge-coupled device camera (Leica Microsystems, Wetzlar, Germany).

Magnetic Resonance Imaging

Mice were anesthetized with isoflurane in oxygen and nitrous oxide at 3% for induction and 1-2% for maintenance to maintain a respiratory rate between 80 and 100 breaths per minute measured with a pillow sensor under the abdomen. Mice were settled on a circulating warm water blanket to maintain normal body temperature. Magnetic resonance imaging (MRI) and 3D time-of-flight magnetic resonance angiography (MRA) were performed using a 7 Tesla small-animal, 30-cm horizontal-bore magnet and a BioSpec Avance III spectrometer (Bruker Corp., Billerica, MA, USA) with a 116-mm high-power gradient set (600 mT/m) and a 30-mm whole-body mouse quadrature coil. Fast spin-echo scout images of the brain were acquired in three orthogonal planes (repetition time [TR] 1000 msec, echo time [TE] 12.5 msec, effective TE [TEeff] 50 msec, 128 × 128 matrix, 0.234 × 0.234 × 1.5–mm voxels, number of acquisitions [NEX] 1) and were loaded as a reference to determine the optimal positioning of the T2-weighted and MRA sequences. Brain T2-weighted images were acquired through rapid acquisition with relaxation enhancement in the coronal plane (TR 5000 msec, TE 12 msec, TEeff 60 msec, Rare Factor 8, 180 × 180 matrix, 0.1 × 0.1 × 0.5–mm voxels, 30 slices, NEX 4). Three-D time-of-flight MRA images were acquired using the flow-compensated gradient echo method (TR 30 msec, TE 3.5 msec, $\alpha = 30°$, 180 × 180 × 200 matrix, 0.1 × 0.1 × 0.1–mm voxels, NEX 4) with the addition of magnetization transfer pulses for additional background signal suppression (offset frequency = 1500 Hz, radiofrequency amplitude = 0.5 μT). Data were

acquired with ParaVision 360 software, version 5.1 (Bruker Corp.), and images were processed with ImageJ software, version 1.53t.[6]

Free Floating Brain Sections

Mice were anesthetized intraperitoneally with ketamine hydrochloride (100 mg/kg body weight) and xylazine (10 mg/kg body weight). They were sequentially perfused with PBS containing 20% heparin (50 units/mL) and 4% paraformaldehyde in 0.1 M phosphate buffer (PB) (pH 7.4). After overnight fixation in 4% paraformaldehyde at 4°C, brains were washed with PBS and cryopreserved in 30% sucrose for 48 hours. Brains were sectioned coronally with a Leica VT 1000S vibratome (Leica Microsystems, Wetzlar, Germany) set to 40 µm section intervals.

Immunofluorescence Staining

Mouse brain tissue sections were blocked in 0.1 M PB containing 10% donkey serum and 0.2% Triton X-100 for 2 hours at room temperature and incubated in primary antibodies diluted in 0.1 M PB containing 2% donkey serum and 0.2% Triton X-100 overnight at 4°C. The following primary antibodies were used: CD31 (1:50, Biocare Medical, Pacheco, CA, USA, catalog number: CM303B), CD68 (1:100, Bio-Rad, Hercules, CA, USA, catalog number: MCA1957T), and alpha smooth muscle–actin clone 1A4 (1:100, Sigma-Aldrich, St. Louis, MO, USA, catalog number: C6198). Sections were washed with 0.1 M PB and incubated in fluorescent dye-conjugated secondaries antibody diluted in 0.1 M PB containing 2% donkey serum and 0.2% Triton X-100 for 2 hours at 4°C. Secondary antibodies included Alexa Fluor 488-conjugated donkey anti-rat (1:100, Invitrogen, Waltham, MA, USA, catalog number: A21208) and Alexa-Fluor 647-conjugated donkey anti-rat (1:100, Invitrogen, catalog number:

A48272). After nuclear immunofluorescent staining with 4′,6-diamidino-2-phenylindole (DAPI, 1:1000, Sigma-Aldrich, catalog number: D9542), tissue sections were mounted on gelatin-coated slides using Prolong Gold Antifade Reagent (Thermo Fisher Scientific, Waltham, MA, USA). Images were captured with the Nikon Eclipse Ti2 Confocal Microscope (Nikon Corp., Minato City, Tokyo, Japan).

Prussian Blue Staining

Prussian blue staining was performed using the Iron Stain Kit (Abcam, Cambridge, UK, catalog number: ab150674) according to the manufacturer’s instructions. Briefly, mouse brain sections were equilibrated in distilled water and incubated with potassium ferrocyanide and hydrochloric acid (1:1) for 5 minutes. The sections were mounted with Prolong Gold Antifade Reagent (Thermo Fisher Scientific, catalog number: P36930) and imaged with the MZ8 stereomicroscope (Leica Microsystems).

Statistical Analysis

All data were stored and analyzed in a repository created with Microsoft Excel, version 16.43 (Microsoft Corp., Seattle, WA). For the statistical analysis, the Student’s *t* test or the one-way analysis of variance (ANOVA) with Tukey’s posthoc test for multi-group comparisons was applied in GraphPad Prism, version 9.3.1 (GraphPad Software, La Jolla, CA, USA). Continuous variables are presented as mean and standard deviation. The level of significance was set at $p<0.05$.

REFERENCES

1. Park SO, Lee YJ, Seki T, Hong K-H, Fliess N, Jiang Z, et al. Alk5-and tgfbr2-independent role of alk1 in the pathogenesis of hereditary hemorrhagic telangiectasia type 2. 2008;111:633-642
2. Olivetti PR, Lacefield CO, Kellendonk C. A device for stereotaxic viral delivery into the brains of neonatal mice. *BioTechniques*. 2020;69:307-312
3. Chen SY, Kuo HY, Liu FC. Stereotaxic surgery for genetic manipulation in striatal cells of neonatal mouse brains. *Journal of visualized experiments : JoVE*. 2018
4. Davidson S, Truong H, Nakagawa Y, Giesler GJ, Jr. A microinjection technique for targeting regions of embryonic and neonatal mouse brain in vivo. *Brain Res*. 2010;1307:43-52
5. Hao Q, Zhu Y, Su H, Shen F, Yang G, Kim H, et al. Vegf induces more severe cerebrovascular dysplasia in endoglin than in alk1 mice. 2010;1:197-201
6. Schneider CA, Rasband WS, Eliceiri KW. Nih image to imagej: 25 years of image analysis. *Nature methods*. 2012;9:671-675

Supplemental Table 1. Stereotaxic coordinates for intracerebral injection of neonatal mice on postnatal day 1

Mouse brain target region	*X* coordinate[1] (mm)	*Y* coordinate[2] (mm)	*Z* coordinate[3] (mm)
Striatum	+ 2.4	± 1.0	– 1.7
Parietal cortex	+ 0.5	± 1.5	– 0.7
Cerebellum	– 1.5	± 0.0	– 1.8

[1]Values refer to distance (mm) from lambda; positive (+) values indicate anterior direction; negative values (–) indicate posterior direction.
[2]Values refer to distance (mm) from midline; positive (+) values indicate right direction and negative values (–) indicate left direction.
[3]Values refer to depth (mm) from surface of skin.

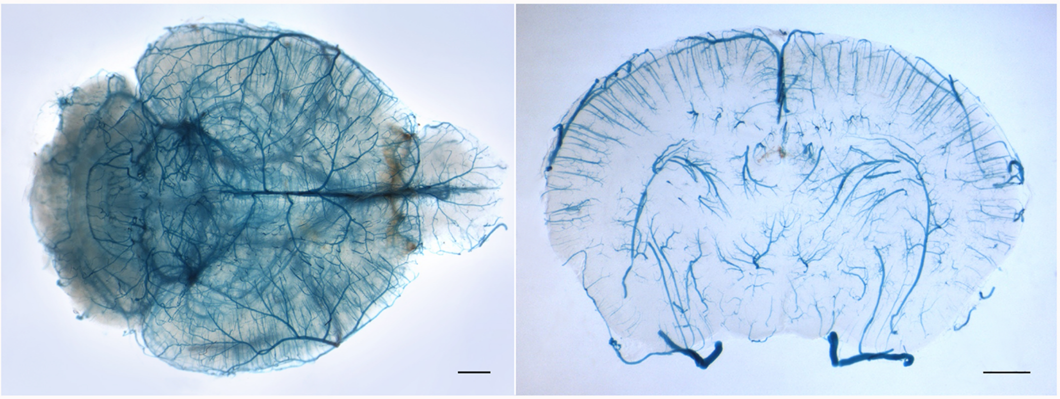

Supplemental Figure 1. Microscopy imaging of a latex-dye perfused brain showing normal cerebrovasculature after stereotaxic injection of 4-hydroxytamoxifen in a control ($R26^{CreER/-}$;$\textit{Alk1}^{2f/2f}$) mouse. Whole brain (left) and coronal section (right) images. Bar, 1 mm.

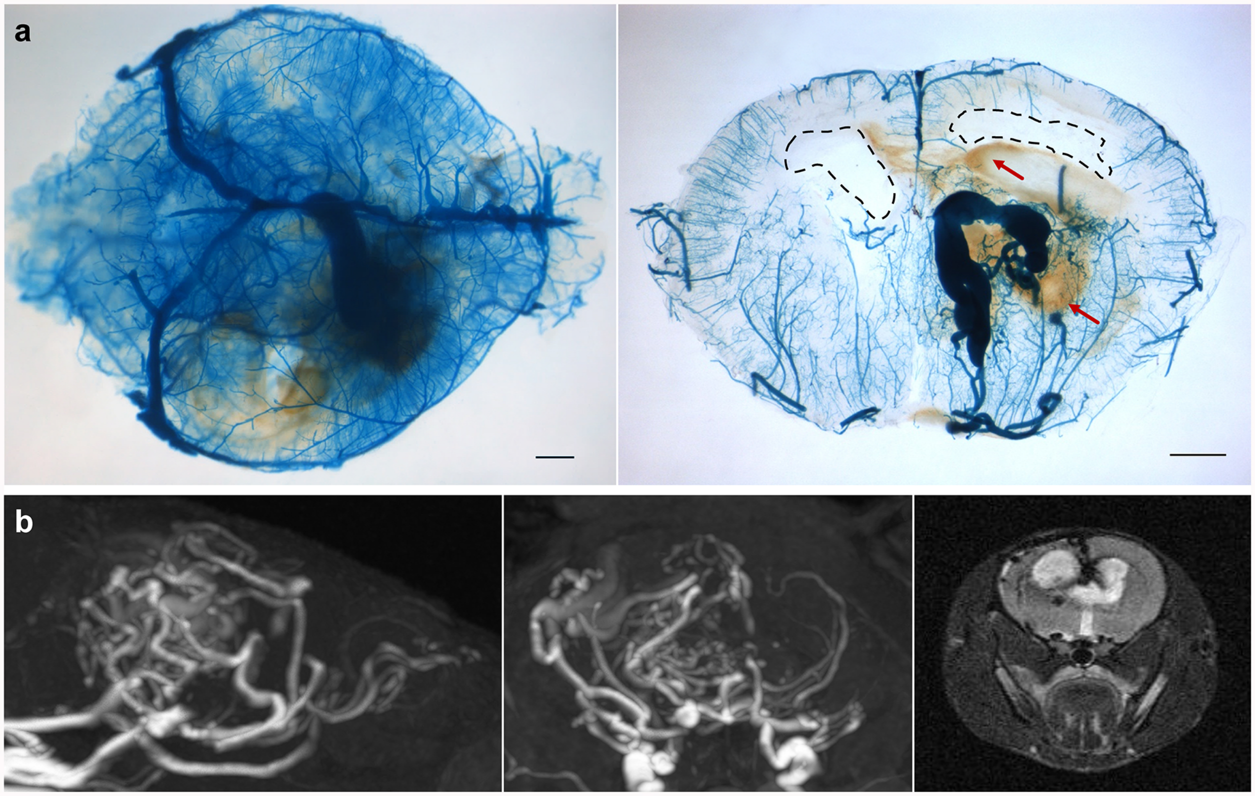

Supplemental Figure 2. Imaging studies of a hydrocephalic mouse brain after stereotaxic intracerebral injection of 4-hydroxytamoxifen in *Alk1*-inducible knockout mice. (**a**) Microscopy images showing a latex-dye-perfused brain with a brain AVM in the right striatum. Whole brain (left) and coronal section (right) images. *Dashed black outlines* indicate enlarged ventricles, and r*ed arrows* indicate associated hemorrhage. (**b**) 3-dimensional time-of-flight magnetic resonance angiography (MRA) images showing sagittal (*left*) and coronal (*center*) views and T2-weighted magnetic resonance image (MRI) image showing a coronal view (*right*) of a large nidal brain AVM in the left parietal cortex. Bar, 1 mm.

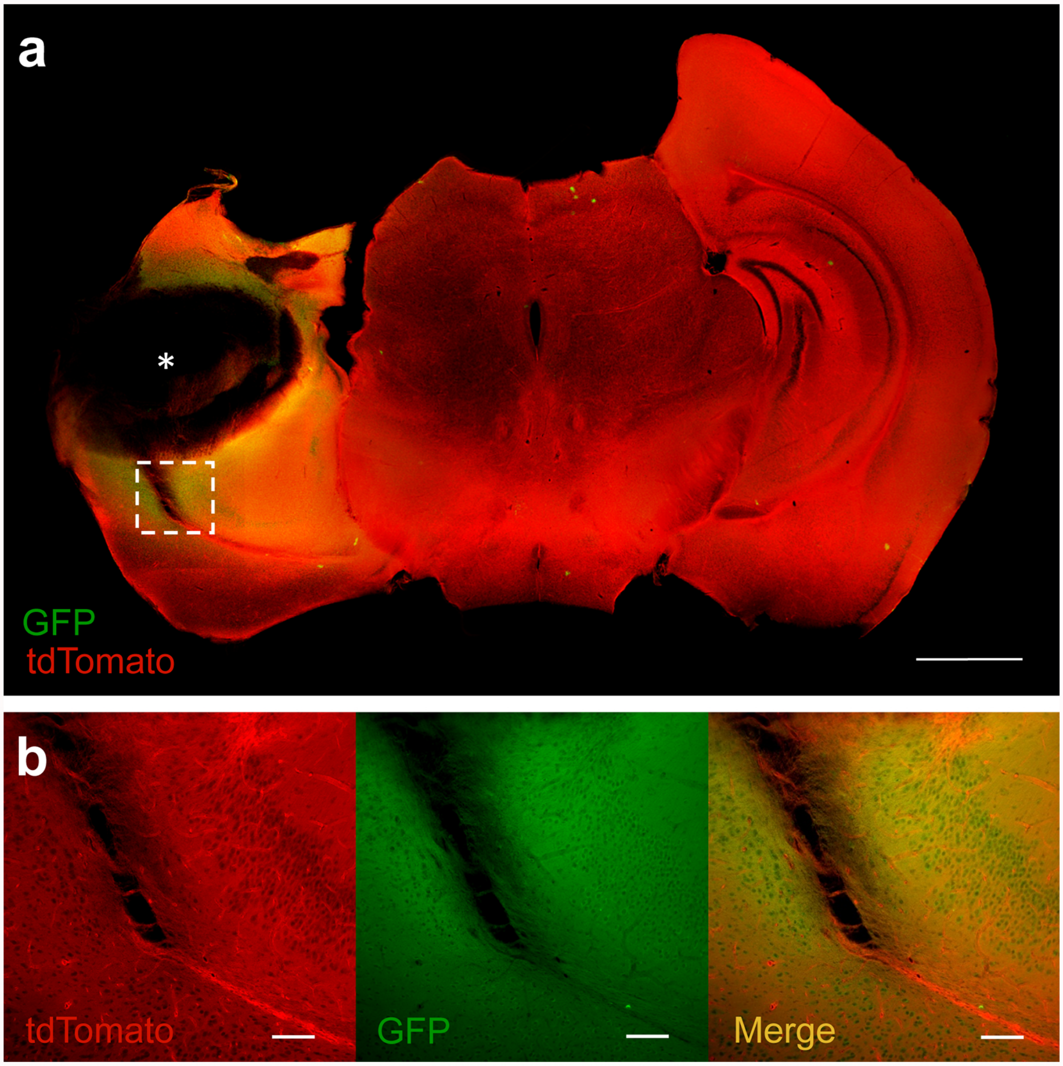

Supplemental Figure 3. Validation of localized Cre activation in R26$^{CreER/mTmG}$;*Alk1*$^{2f/2f}$reporter mice. (**a**) Fluorescence image of a coronal mouse brain tissue section showing mG labeling (GFP fluorescence) circumferential to a nidal brain AVM in the left-hemispheric parietal cortex, which correlates with the stereotaxic intracerebral injection of 4-hydroxytamoxifen into the left parietal cortex. *Location of brain AVM. *White outline* indicates area of magnification. Bar, 1000 μm. (**b**) Magnified views of mT labeling (TdTomato), mG labeling (GFP), and merged mT/mG labeling. Bar, 100 μm.

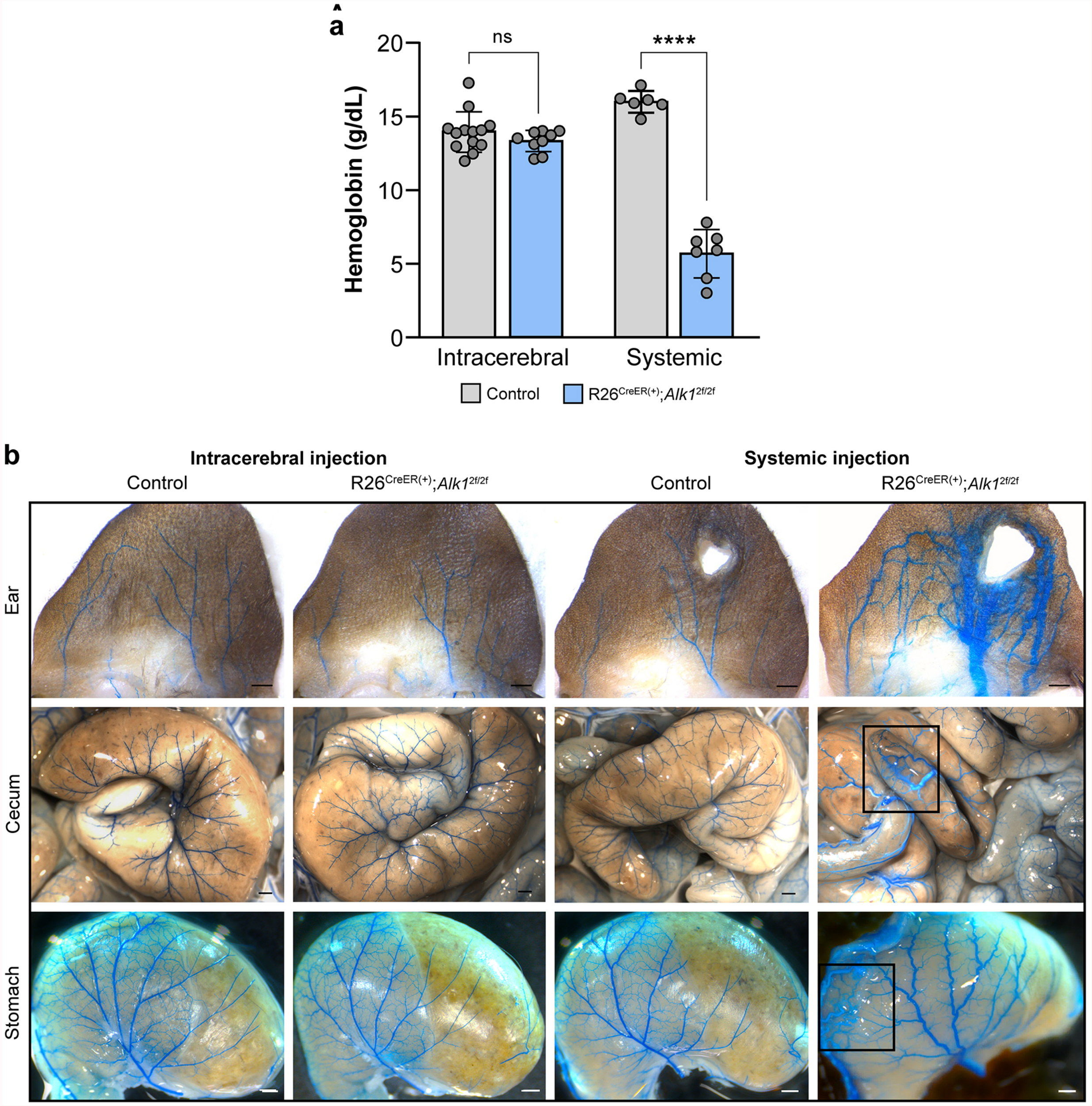

Supplemental Figure 4. Comparison of localized and systemic Cre activation in *Alk1*-inducible knockout mice. (**a**) Scatter plot bar graph representing hemoglobin levels of control ($R26^{+/+}$;$Alk1^{2f/2f}$, gray) and mutant ($R26^{CreER/+}$;$Alk1^{2f/2f}$, blue) mice after localized and systemic activation of Cre. Mean (SD), ANOVA. ****p<0.001; ns, not significant. (**b**) Microscopy images show arteriovenous malformations in control and mutant mouse ears, stomachs, and ceca. Bar, 1 mm.
